# Macular Pigment Optical Density in Chinese Primary Open Angle Glaucoma Using the One-Wavelength Reflectometry Method

**DOI:** 10.1155/2016/2792103

**Published:** 2016-04-10

**Authors:** Yuying Ji, Chengguo Zuo, Mingkai Lin, Xiongze Zhang, Miaoling Li, Lan Mi, Bing Liu, Feng Wen

**Affiliations:** State Key Laboratory of Ophthalmology, Zhongshan Ophthalmic Center, Sun Yat-sen University, Guangzhou 510060, China

## Abstract

*Purpose*. To investigate macular pigment optical density (MPOD) and its relationship with retinal thickness in primary open angle glaucoma (POAG) patients using the one-wavelength reflectometry method.* Methods*. A total of 30 eyes from 30 POAG patients (18 males and 12 females, mean age 47.27 ± 16.93) and 52 eyes from 52 controls (27 males and 25 females, mean age 49.54 ± 19.15) were included in this prospective, observational, case-control study. MPOD was measured in a 7-degree area using one-wavelength reflectometry method. Two parameters, max and mean optical density (OD), were used for analyses. Spectral-domain-optical coherence tomography was used to measure retinal thickness, including central retinal thickness (CRT), the macular ganglion cell complex (GCC), and the circumpapillary retinal nerve fiber layer (RNFL).* Results*. Both maxOD and meanOD were significantly reduced in POAG patients compared with normal subjects (*P* < 0.001). GCC, CRT, and RNFL thicknesses were also significantly reduced in POAG patients (*P* < 0.001). GCC thickness had a positive relationship with MPOD.* Conclusions*. MPOD within the 7-degree area was significantly lower in Chinese POAG patients than in control subjects, and GCC thickness was significantly and positively associated with MPOD. Whether the observed lower MPOD in POAG contributes to the disease process or is secondary to pathological changes caused by the disease (such as loss of ganglion cells) warrants further and longitudinal study.

## 1. Introduction

Macular pigment (MP) is composed of lutein, zeaxanthin, and mesozeaxanthin. MP has a peak distribution in the fovea of the retina. The concentration of xanthophylls in the peripheral retinal is 100-fold less than that of the fovea [1]. Lutein and zeaxanthin are located in the Henle fiber layer, the inner retinal layer, and the rod outer segment in mature retinal tissues [2–4]. MP protects the retina by filtering blue light and quenching singlet oxygen [5, 6]. Two categories of methods are primarily available to measure macular pigment optical density (MPOD): the psychological technique and objective technique [7]. The one-wavelength reflectometry method is a new objective method covering a 7-degree area that contains the majority of MP [2, 8]. Potential factors associated with MPOD include age, sex, body mass index (BMI), and smoking status [9–12].

Glaucoma is an optic neuropathy characterized by retinal ganglion cell death and can result in irreversible and progressive vision and visual field loss which affects millions of people worldwide. Retinal nerve fiber layer (RNFL) measurements and visual field tests are the most commonly used methods to estimate and evaluate the extent of the disease. The nerve fiber layer, ganglion cell layer, and inner plexiform layer constitute the ganglion cell complex (GCC), corresponding to the axons, cell bodies, and dendrites of the retinal ganglion cell, respectively [13]. Researchers have demonstrated some macular parameters where GCC exhibit similar diagnostic powers compared with peripapillary RNFL parameters [14–16].

Limited studies have investigated MPOD in glaucoma. Recently two studies have shown that MPOD in open angle glaucoma patients is significantly reduced compared with normal people using the psychophysical method within 1-degree area [17, 18]. MPOD in Chinese primary open angle glaucoma (POAG) patients has not been investigated yet using the one-wavelength reflectometry method. We conducted this study to verify whether MP is lower in Chinese primary open angle glaucoma (POAG) patients using the one-wavelength reflectometry method and to observe the correlations between MPOD and demographic and retinal thickness factors.

## 2. Materials and Methods

This is a prospective, observational, case-control study. The study adhered to the tenets of the declaration of Helsinki. The Institutional Review Board of Zhongshan Ophthalmic Center approved this research, and all participants provided written informed consent.

### 2.1. Subjects

Patients were recruited from the glaucoma outpatient department in Zhongshan Ophthalmic Center. The control group consisted of volunteers from the outpatient department with normal results upon ocular examination. POAG was defined as adult onset, with an open, normal-appearing anterior chamber angle and typical optic nerve head damage and/or glaucomatous visual field damage without other known explanations. All participants underwent a detailed examination, including visual acuity, slit lamp biomicroscopy, direct ophthalmoscopy, optometry, and noncontact tonometry. The cup to disc ratio (C/D) was recorded. Exclusion criteria included a best-corrected visual acuity less than 63 letters using the Early Treatment Diabetic Retinopathy (ETDRS) chart, cornea disease, cataract, artificial lens, refractive error between −6.0 D and +6.0 D, fundus disease, any disease that may influence the refracting media of the eye (e.g., severe vitreous opacities), uncontrolled hypertension, and a medical history that may influence the absorption of xanthophylls such as lutein supplementation.

Other demographic data were also collected including age, gender, height, weight, and smoking status. Body mass index (BMI) was calculated by dividing weight in kilograms by height in meters squared.

### 2.2. MPOD Measurement

A one-wavelength fundus reflectance method (Visucam 200; Carl Zeiss Meditec) was used for detection of MPOD as previously described [12]. The right or left eye was randomly selected for measurement. All subjects' pupils were dilated to a minimum diameter of 7 mm using 1% tropicamide. The parameters and profiles of MPOD in a 7-degree eccentricity that corresponded to a 4 mm diameter were evaluated and output. Parameters included max and mean optical density (OD), volume, and area. MaxOD and meanOD with units “d.u.” (initial of density units) were used for the analyses.

### 2.3. Optical Coherence Tomography (OCT) Measurement

All subjects underwent a spectral-domain-OCT examination (SD-OCT, OSE-200, MOPTIM, Shenzhen, China). We acquired GCC thickness and central retinal thickness (CRT) measurements using the 6-line scan. This scan protocol was centered on the fovea and consisted of 29000 A-scans over a 6 mm circle area with three concentric circles with diameters of 1 mm, 3 mm, and 6 mm, respectively. We used the central 6 mm area for the analysis including total, superior, inferior, nasal, and temporal area in OCT. The distance from the internal limiting membrane and outer edge of the outer plexiform layer was defined as GCC thickness. CRT thickness was defined as distance between the internal limiting membrane and the inner edge of the retinal pigment epithelium. Outer retinal (OR) thickness was calculated by subtracting GCC thickness from CRT. Circumpapillary RNFL scans were obtained using the standard 3.4 mm 12-degree circumpapillary nerve fiber layer scan protocol. We recorded superior, inferior, nasal, temporal, and total RNFL thickness.

### 2.4. Visual Field Test and Disease Severity

All POAG patients underwent the visual field test using the 30-2 Threshold Test on the Humphrey Visual Field Analyzer (Carl Zeiss Meditec, Jena, Germany). According to the mean deviation (MD) values generated by the software, three groups of disease severity were classified: the mild group with MD > −6 dB, the moderate group with MD between −6 and −12 dB, and the severe group with MD < −12 dB.

### 2.5. Statistical Analysis

Data were processed and analyzed using SPSS 20.0 software (Inc., Chicago, IL, USA). All continuous variables were presented as the mean ± standard deviation (SD). Fisher's exact test or Chi-square test was used for the analysis of categorical variables. Two independent samples *t*-test was used to assess the differences between the two groups. Pearson correlation coefficient was used to estimate the relationships between MPOD and retinal thickness in all subjects. Multiple linear regression was used to evaluate the relationship among demographic factors, retinal thickness, and MPOD. *P* < 0.05 was considered statistically significant.

## 3. Results

A total of 30 eyes from 30 POAG patients and 52 eyes from 52 normal participants were included in the study. The participants were all from the Chinese Han population.  Table 1 presents the basic characteristics of the subjects. No differences in age, sex, BMI, and smoking status were noted between the two groups. The cup to disc ratio was 0.83 ± 0.15 in POAG group and 0.32 ± 0.12 in the control group.

### 3.1. MPOD in the POAG and Control Groups

In the POAG group, maxOD was 0.301 ± 0.076 d.u. and meanOD was 0.116 ± 0.033 d.u. In the control group, maxOD was 0.370 ± 0.056 d.u. and meanOD was 0.137 ± 0.026 d.u. MPOD in the glaucoma group was significantly reduced compared with the control group (Table 2).

### 3.2. OCT Measurements of the Glaucoma and Control Groups

OCT measurements values are presented in Table 3. Compared with the control groups, GCC, CRT, and RNFL thicknesses were significantly thinner in the glaucoma groups (all *P* values < 0.001). No significant difference in OR thickness was noted between the two groups (*P* > 0.05).

### 3.3. Correlation between MPOD and Retinal Thickness

Table 4 presents the Pearson correlation results. The inferior, temporal, and total GCC thickness positively correlated with maxOD in POAG patients (*P* = 0.004, *P* = 0.003, and *P* = 0.020, resp.). A positive relationship also existed between inferior outer retinal thickness and maxOD and meanOD in POAG patients (*P* = 0.012, *P* = 0.035, resp.). No significant correlations were noted between retinal thickness parameters and MPOD in the control group. RNFL did not correlate with MPOD in the POAG or control group.

**Figure 1:**
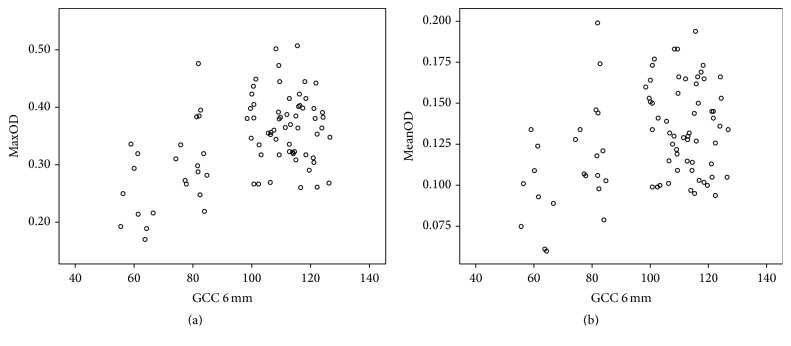
Scatter plot depicting the correlation between GCC thickness and MPOD. (a) Positive relation between maxOD and GCC. (b) Positive relation between meanOD and GCC.

**Figure 2:**
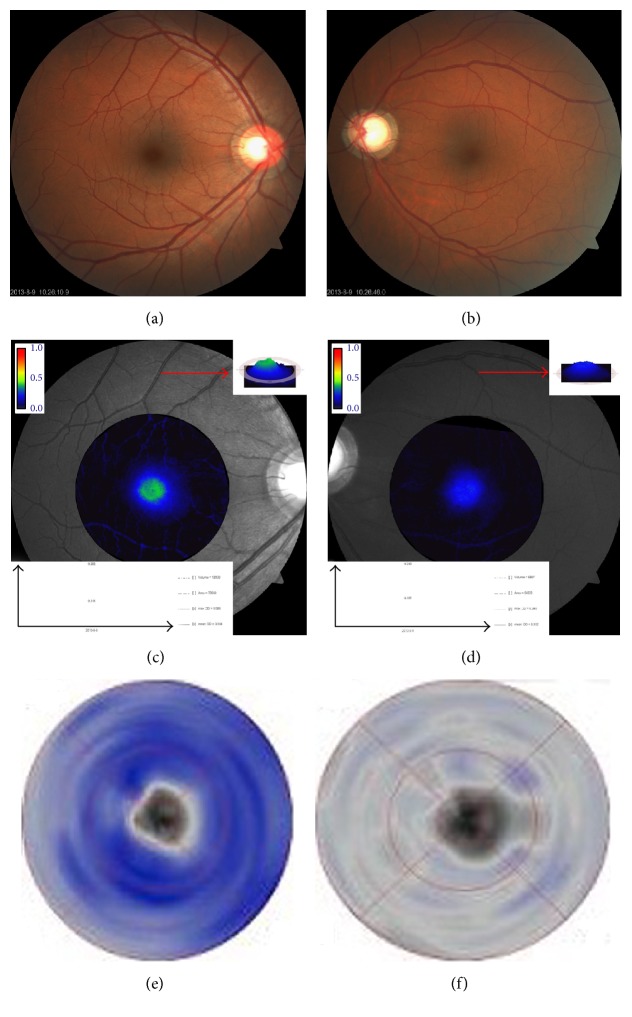
Patient examples of MPOD and GCC. (a) Fundus photography of case 1 with a cup to disc ratio of approximately 0.6. (b) Fundus photography of case 2 with a cup to disc ratio of approximately 0.9. (c and d) MPOD profile indicating that case 1 (c) exhibits a higher MPOD than case 2 (d) (red arrow). (e and f) GCC profile indicating that case 1 (e) exhibits a thicker GCC than case 2 (f) (deeper blue means thicker thickness).

Table 5 presents the multiple linear regression results. Age and BMI significantly correlated with maxOD and meanOD (for age, *P* < 0.001, and for BMI, *P* = 0.042, *P* = 0.028, resp.). Furthermore, GCC thickness is positively related to MPOD (for maxOD, *P* < 0.001, and for meanOD, *P* = 0.001).  Figure 1 presents a scatter plot depicting the relationship in a direct manner.  Figure 2 includes classic examples of patients. Patients in the right column had smaller cup to disc ratios, higher MPOD values, and deeper GCC thickness color than patients in the left column.

### 3.4. Correlation between MPOD and MD in POAG Patients

Pearson correlation revealed that no significant relation exists between MPOD values and MD (for maxOD, *P* = 0.876, and for meanOD, *P* = 0.630).

## 4. Discussion

The study was designed to investigate the distribution of MP in Chinese POAG patients using an objective, one-wavelength reflectometry method and to explore the possible associations between MPOD levels and POAG indices. To the best of our knowledge, no previous study has employed this objective method to explore the MPOD in Chinese POAG patients.

The present study found that MPOD in POAG patients was significantly lower than normal individuals after adjustment for age, BMI, and smoking status. These results are consistent with a previous study conducted in Ireland [17, 18]. It strengthened the fact that MPOD did decrease in glaucoma. Furthermore, in Asian POAG patients, MPOD exhibited the same tendency for change as in Caucasian individuals. The study also found that GCC thickness and RNFL thickness were statistically reduced in POAG patients. Further analysis by Pearson correlation, multiple linear regression, and scatter plot indicated that GCC thickness was positively associated with MPOD. The result was similar to that reported for 88 open angle glaucoma patients that demonstrated that eyes with foveal involvement exhibited lower MPOD than eyes with no foveal involvement [18].

Two possibilities potentially explain why MPOD is reduced in POAG patients. First, lower MPOD values contribute to the risk of disease. Lutein quenches the active oxygen [5]. Numerous studies have shown that oxidative stress is involved in the process of glaucoma [19]. It is hypothesized that oxidative stress plays an early role in the process of glaucomatous optic neuropathy [20]. Oxidative DNA damage is statistically increased in POAG patients compared with normal individuals and antioxidant enzymes are significantly reduced in both blood and serum samples [21–23]. Thus, individuals with lower MPOD values have weaker antioxidant defenses against the glaucomatous process and are more likely to develop glaucoma. Second, loss of “housing” for MP due to loss of the retinal nerve fiber layer may explain reduced MPOD in POAG patient. In addition to the main distribution in the outer plexiform layer of the fovea, MP is also located in the inner retinal layer of the parafoveal intracellular [2, 3]. In glaucoma patients, retinal ganglion cells apoptosis and loss of the retinal nerve fiber layer caused reduced MP localization and a reduction in MP.

Regardless of the cause, supplementation should be advantageous. MP plays an important role in improving glare disability and photo stress recovery. In addition, lutein and zeaxanthin supplementation can improve visual performance in glares [24]. MP can also increase contrast sensitivity [25, 26]. Both of these issues are major problem in POAG patients. Numerous studies suggest that POAG patients had poor performance on many psychophysical tests especially contrast sensitivity and glare disability [27, 28].

The one-wavelength technique we adopted in this study calculated MPOD based on a fundus image generated by a single 460 nm wavelength. The technique is simple and objective with good reproducibility [8, 29]. However, some limitations were also noted. Stray light through an aging lens impacted on the results of the reflectometry method, and a strong cataract impact was noted with the one-wavelength method [7, 8, 30]. Several measures were obtained to minimize the impact. The cataracts were excluded. The ages of POAG patients and normal controls were matched to each other. In multiple linear regression, age was adjusted. Furthermore, the intensity of the nerve fiber layer has an impact on the reflectance [31]. Thus, thinner GCCs would produce less reflectance and a lower MPOD using reflectometry method.

There were some limitations of our study. We analyzed a small sample size without further classification and periodization of glaucoma. Further investigations to explore whether MP is related to the disease are expected.

In conclusion, using the one-wavelength reflectometry method, POAG patients had lower MPOD in this Chinese cohort. GCC thickness was positively related to MPOD. A further study exploring the causal relationship between MPOD and glaucoma is needed.

## Figures and Tables

**Table 1:** Characteristics of the study eyes.

	POAG (*n* = 30)	Control (*n* = 52)	*P*
Mean age ± SD (y) (range)	47.27 ± 16.93 (20–76)	49.54 ± 19.15 (10–77)	0.413
Sex, *n* (male/female)	18/12	27/25	0.500
Smoking, *n* (yes/no)	7/23	5/47	0.112
BMI ± SD (range)	22.24 ± 2.46 (19.10–28.32)	21.24 ± 2.97 (15.31–28.60)	0.086
C/D ± SD	0.83 ± 0.15	0.32 ± 0.12	<0.001

BMI: body mass index.

C/D: cup to disc ratio.

**Table 2:** Comparison of macular pigment optical density values in two groups.

	POAG		Control		*P*
	Mean	SD	Mean	SD	
MaxOD (d.u.)	0.301	0.076	0.370	0.056	<0.001
MeanOD (d.u.)	0.116	0.033	0.137	0.026	<0.001

d.u.: (density units) the unit for maxOD and meanOD.

**Table 3:** Retinal thickness values and comparison of study subjects.

	POAG		Control		*P*
	Mean, *µ*m	SD, *µ*m	Mean, *µ*m	SD, *µ*m	
GCC					
6 mm	80.80	17.33	113.10	7.71	<0.001
Superior	82.92	18.47	116.95	9.00	<0.001
Inferior	80.89	19.10	116.40	8.16	<0.001
Nasal	86.01	21.66	119.71	9.43	<0.001
Temporal	77.65	13.12	96.91	7.22	<0.001
CRT					
6 mm	290.86	26.74	328.05	17.42	0.021
Superior	293.98	29.47	333.12	18.03	<0.001
Inferior	285.61	28.80	327.45	19.86	<0.001
Nasal	299.05	30.58	338.07	19.81	0.001
Temporal	288.80	26.54	320.88	16.76	<0.001
OR					
6 mm	210.06	15.40	214.95	12.64	0.271
Superior	211.06	17.74	216.18	12.81	0.135
Inferior	204.72	14.83	211.05	14.69	0.065
Nasal	213.04	16.78	218.35	15.74	0.155
Temporal	211.15	19.66	223.97	13.90	0.308
RNFL					
Total	70.20	10.84	97.93	8.33	<0.001
Superior	78.25	15.74	113.71	16.95	<0.001
Inferior	78.01	21.06	128.71	16.59	<0.001
Nasal	58.00	8.47	68.38	6.57	<0.001
Temporal	66.53	11.23	81.42	10.60	<0.001

GCC: ganglion cell complex.

CRT: central retinal thickness.

OR: outer retinal thickness (CRT minus GCC).

RNFL: retinal nerve fiber layer.

**Table 4:** Pearson correlations between MPOD and retinal thickness in the POAG and control groups.

	POAG				Control			
	MaxOD		MeanOD		MaxOD		MeanOD	
	*R*	*P*	*R*	*P*	*R*	*P*	*R*	*P*
GCC								
6 mm	0.423	**0.020**	0.266	0.155	−0.150	0.289	−0.141	0.318
Superior	0.334	0.071	0.174	0.357	−0.098	0.490	−0.101	0.476
Inferior	0.509	**0.004**	0.355	0.054	−0.225	0.109	−0.189	0.181
Nasal	0.342	0.065	0.207	0.272	−0.153	0.278	−0.131	0.356
Temporal	0.521	**0.003**	0.373	0.037	0.038	0.789	0.038	0.790
OR								
6 mm	0.359	0.051	0.113	0.551	−0.081	0.570	−0.138	0.330
Superior	0.212	0.261	0.144	0.449	−0.159	0.261	−0.201	0.152
Inferior	0.451	**0.012**	0.387	**0.035**	0.007	0.961	−0.057	0.688
Nasal	0.321	0.083	0.281	0.133	−0.023	0.869	−0.092	0.516
Temporal	0.333	0.073	0.249	0.185	−0.216	0.124	−0.252	0.071
RNFL								
Total	0.236	0.210	0.113	0.551	0.020	0.891	0.002	0.987
Superior	0.027	0.886	−0.046	0.809	0.022	0.875	−0.020	0.890
Inferior	0.212	0.262	0.112	0.557	0.107	0.449	0.090	0.525
Nasal	0.181	0.338	0.088	0.642	−0.196	0.164	−0.134	0.344
Temporal	0.338	0.068	0.226	0.230	0.107	0.450	0.071	0.617

GCC: ganglion cell complex.

OR: outer retinal thickness (values of CRT subtracting GCC).

RNFL: retinal nerve fiber layer.

**Table 5:** Multiple linear regression model showing the relationship between age, sex, BMI, retinal thickness, maxOD, and meanOD.

Variables	MaxOD		MeanOD	
	*β*	*P*	*β*	*P*
Age	0.408	**<0.001**	0.591	**<0.001**
Sex	0.167	0.089	0.172	0.066
BMI	−0.188	0.042	−0.193	0.028
smoking	0.030	0.763	0.047	0.622
GCC	0.454	**<0.001**	0.316	**0.001**
OR	0.029	0.763	0.010	0.909

BMI: body mass index.

GCC: ganglion cell complex.

OR: outer retinal thickness (CRT minus GCC).
